# Репродуктивная функция женщин после радиойодтерапии дифференцированного рака щитовидной железы

**DOI:** 10.14341/probl13407

**Published:** 2024-04-24

**Authors:** М. О. Корчагина, Е. Н. Андреева, М. С. Шеремета, Г. А. Мельниченко

**Affiliations:** Национальный медицинский исследовательский центр эндокринологии; Национальный медицинский исследовательский центр эндокринологии; Российский университет медицины; Национальный медицинский исследовательский центр эндокринологии; Национальный медицинский исследовательский центр эндокринологии

**Keywords:** дифференцированный рак щитовидной железы, терапия радиоактивным йодом, осложнения, репродуктивная функция, овариальный резерв, фертильность

## Abstract

Дифференцированный рак щитовидной железы (ДРЩЖ) — наиболее распространенное злокачественное новообразование эндокринной системы. ДРЩЖ составляет 90–95% случаев рака щитовидной железы (ЩЖ) и преобладает среди женского населения во всех возрастных группах. Хирургическое вмешательство — стандартный метод лечения пациентов с ДРЩЖ, за которым по показаниям следует терапия радиоактивным йодом (РЙТ). После основного лечения пациенты получают терапию тиреоидными гормонами в различном режиме — в виде заместительной или супрессивной терапии.

На протяжении многих лет изучают влияние РЙТ на здоровье пациентов c ДРЩЖ. Были выявлены вторичные осложнения, обусловленные накоплением 131I в органах и тканях, а также непосредственным участием некоторых органов в метаболизме соединений, содержащих 131I, и его выведении. В связи с тем, что ДРЩЖ нередко выявляют у молодых пациенток, репродуктивное здоровье на фоне РЙТ также стало предметом многочисленных исследований. Настоящий обзор литературы обобщает данные о влиянии РЙТ на репродуктивное здоровье женщин с ДРЩЖ, что позволяет определить траекторию для дальнейших исследований в этой области и необходимость изменения тактики ведения пациенток, в том числе с учетом планирования беременности.

## Abstract (en)

Differentiated thyroid carcinoma (DTC) is the most common endocrine cancer, accounting for 90–95% of thyroid malignancy and predominant in the female population of all age groups. Surgery is the standard primary treatment for patients with DTC, followed by radioactive iodine therapy (RAIT) when indicated. After the main treatment, patients receive thyroid hormone therapy in a variety of regimens, either as a replacement therapy or as a therapy intended to suppress secretion of thyroid-stimulating hormone (TSH).

The impact of RAIT on the health of individuals with DTC has been studied for years. The accumulation of 131I in organs and tissues, as well as some organs’ direct involvement in the metabolism of 131I-containing substances and its excretion, can lead to secondary complications. Reproductive health in the context of RAIT has also been a popular research topic because DTC is frequently found in young individuals.

We may identify a direction for future study in this field and the requirement for adjustments in patient management thanks to the data summarized in this review on the effect of RAIT for DTC on female reproductive health.

## ВВЕДЕНИЕ

Рак щитовидной железы (РЩЖ) — наиболее распространенное злокачественное новообразование эндокринной системы. По данным Международного агентства по изучению рака, заболеваемость РЩЖ в Российской Федерации на 2020 г. составила 10,7 случая на 100 000 женщин (5-е место среди злокачественных новообразований различных локализаций у женщин) и 2,6 случая на 100 000 мужчин (20-е место среди злокачественных новообразований различных локализаций у мужчин) [1]. Дифференцированный РЩЖ (ДРЩЖ) — самый часто встречаемый вариант РЩЖ, представляющий собой карциному из фолликулярных клеток. Папиллярный и фолликулярный РЩЖ — наиболее распространенные гистотипы ДРЩЖ, встречающиеся приблизительно в 80 и 15% случаев соответственно [2]. В последние десятилетия благодаря улучшению методов диагностики возросла выявляемость ДРЩЖ. Как правило, новые случаи представлены папиллярным раком, причем у женщин он выявляется в 2,5–3 раза чаще, чем у мужчин. Смертность от ДРЩЖ остается низкой, а 5-летняя выживаемость при условии своевременного обнаружения и качественно оказанной медицинской помощи достигает 95% [3].

## ЛЕЧЕНИЕ ДРЩЖ

Хирургическое лечение в различном объеме — стандартный метод лечения ДРЩЖ. В настоящее время такой подход рекомендован с целью повышения выживаемости при IV, V и VI категории (Bethesda Thyroid Classification, 2009) цитологического заключения пунктата, полученного в ходе тонкоигольной аспирационной биопсии (ТАБ) [4]. После хирургического лечения пациенты проходят стадирование послеоперационного риска рецидива на основании рекомендаций Американской тиреоидологической ассоциации (American Thyroid Association, 2015 г.) для определения дальнейшей тактики ведения. В настоящее время использование послеоперационной терапии радиоактивным йодом (РЙТ) при ДРЩЖ осуществляется в соответствии со стратификацией риска рецидива заболевания. РЙТ — неотъемлемая часть лечения пациентов с высоким риском рецидива, улучшающая общую и безрецидивную выживаемость. Также РЙТ в индивидуальном порядке может быть рассмотрена у пациентов промежуточного риска. Посттерапевтическая сцинтиграфия всего тела (СВТ) позволяет выявить наличие и установить объем остаточной тиреоидной ткани, а также обнаружить метастазы ДРЩЖ, способствуя завершению стадирования заболевания и определяя дальнейшее ведение пациента [5]. После основного лечения назначаются тиреоидные гормоны с заместительной или супрессивной целью в зависимости от группы риска рецидива [6][7].

Таким образом, ведение пациентов с ДРЩЖ зависит от результатов цитологического исследования, размеров опухоли, стадии заболевания и рисков рецидива. Стандартная схема комбинированного лечения ДРЩЖ включает тиреоидэктомию (ТЭ), РЙТ и супрессивную терапию.

## ТЕРАПИЯ РАДИОАКТИВНЫМ ЙОДОМ ПО ПОВОДУ ДРЩЖ

В 2022 г. в консенсусе Европейской тиреоидной ассоциации (ETA), Американской тиреоидной ассоциации (ATA), Европейской ассоциации ядерной медицины (EANM) и Общества ядерной медицины и молекулярной визуализации (SNMMI) было определено три цели терапии ¹³¹I: аблация остаточной тиреоидной ткани (как правило, так называют первое введение ¹³¹I), адъювантное лечение, лечение известного заболевания [8]. Для обеспечения эффективности РЙТ пациент проходит специальную подготовку, направленную на повышение уровня тиреотропного гормона (ТТГ) более 30 мЕД/л [9]. ТТГ — главный туморотропный фактор, экспрессия натрий-йодидного симпортера (НЙС), белка — переносчика йода, в основном регулируется им. ТТГ повышает концентрацию НЙС на базолатеральной мембране тиреоцита, что в свою очередь повышает захват радиоактивного йода, обнаружение и деструкцию остаточной тиреоидной ткани или метастатических очагов. Повысить ТТГ можно эндогенно — путем отмены гормонов ЩЖ за 4 недели до РЙТ, тогда пациент находится в состоянии гипотиреоза, и экзогенно — путем введения рекомбинантного человеческого ТТГ (рчТТГ). У обоих методов подготовки есть свои недостатки: первый приводит к развитию клинической картины гипотиреоза, значимо снижая качество жизни пациентов и способствуя нарушению психического и физического здоровья, а один из главных недостатков второго — высокая стоимость [10].

## ВТОРИЧНЫЕ ОСЛОЖНЕНИЯ РЙТ

РЙТ при ДРЩЖ может быть сопряжена с развитием ряда осложнений. Потенциальные вторичные осложнения РЙТ можно разделить на группы в зависимости от времени возникновения/длительности сохранения и от частоты возникновения. У пациентов могут наблюдаться диспепсические явления, дисгевзия, радиационный тиреоидит, реактивные изменения кожи в области ЩЖ, слизистой глотки и гортани, сиалоаденит, облитерация слезоотводящих путей с развитием эпифоры, сухой кератоконъюнктивит, острый гастрит, кариес, лекарственное поражение печени, миелосупрессия, дисфункция яичников (ДЯ), снижение овариального резерва, гипоспермия. Более редкие осложнения включают язвенный цистит, паротит, алопецию, нарушение легочной функции, постлучевой пневмонит, радиационно-индуцированный фиброз легких, вторичный рак [9][11][12].

Наиболее частое раннее осложнение — нарушение работы желудочно-кишечного тракта (ЖКТ), возникающее, как правило, в течение первых 48 часов после РЙТ [13]. На втором месте — боль и отек в области шеи, развивающиеся в те же сроки. Еще одно осложнение — транзиторный сиалоаденит, сопровождающийся припухлостью, дискомфортом, болью в области слюнных желез, чаще околоушных и поднижнечелюстных [14]. Развитие ранних осложнений обусловлено тем, что ¹³¹I или накапливается в органах благодаря наличию в них НЙС, как в случае остаточной ткани ЩЖ и слюнных желез, или выводится ими, как в случае ЖКТ [15]. К факторам риска по развитию ранних осложнений относят высокую активность ¹³¹I (100 мКи и более), длительный гипотиреоз и поражение лимфатических узлов [9][16].

Потенциальные поздние осложнения включают поражение слюнных желез, облитерацию слезоотводящих путей и сухой кератоконъюнктивит, заболевания ЖКТ, поражение легких (у пациентов с легочными метастазами), стойкое подавление функции костного мозга, хроническую азооспермию и гипоспермию, ранее наступление менопаузы и другие [9][17][18]. Одно из наиболее частых поздних осложнений РЙТ по поводу ДРЩЖ — поражение слюнных желез с развитием хронического сиалоаденита и ксеростомии, при этом уже после первой РЙТ наблюдаются изменения в секреторной функции слюнных желез [19–21].

Как правило, существует корреляция между активностью, количеством курсов РЙТ и развитием осложнений: чем выше доза ¹³¹I, получаемая пациентом, тем выше риск развития вторичных осложнений [9][22–25]. Безусловно, ожидаемая польза РЙТ оправдывает риски, связанные с ее применением, однако необходимость в изучении возможных вторичных осложнений, предикторов их развития, а также разработка мер их профилактики неоспорима.

## РЕПРОДУКТИВНАЯ ФУНКЦИЯ ЖЕНЩИН С ДРЩЖ, ПОЛУЧАЮЩИХ РЙТ. ПЕРВЫЕ ИССЛЕДОВАНИЯ

Первые наблюдения о влиянии РЙТ на функцию яичников были сделаны в 1949 г., почти сразу после внедрения ¹³¹I в качестве метода лечения РЩЖ. Trunnell и Marinelli зарегистрировали развитие аменореи, сопровождающейся приливами, у трех женщин репродуктивного возраста с ранее регулярным менструальным циклом (МЦ). Кумулятивные дозы составили 90–458 мКи. Были выдвинуты предположения, что данные осложнения возникли в результате повреждения ткани яичников циркулирующим в крови ¹³¹I, поскольку сами яичники неспособны накапливать ¹³¹I [26]. Похожие наблюдения сделали Dobyns и Maloof: у двух из четырех пациенток 38 лет наблюдалась ДЯ. У одной аменорея развилась после введения 250 мКи ¹³¹I, у другой — после введения 140 мКи, при этом повышение уровня ФСГ в моче соответствовало развитию преждевременной недостаточности яичников (ПНЯ) [27].

Более пристальное внимание изучению функции яичников на фоне РЙТ стало уделяться в 90-х годах прошлого столетия. Raymond и соавт. провели ретроспективное исследование функции яичников у 66 женщин, которые в качестве терапии ДРЩЖ прошли ТЭ и РЙТ. Исследовались уровни ФСГ, ЛГ, пролактина и эстрадиола через 1, 3, 6, 9 и 12 месяцев после РЙТ. В течение первого года после РЙТ у 18 женщин (~30%) развилась транзиторная аменорея, сопровождавшаяся повышением уровня гонадотропинов (P<0,001) и приливами жара, при этом в большинстве случаев аменорея развивалась в течение первых 6 месяцев после РЙТ, но не ранее, чем через месяц. Что интересно, терапия левотироксином натрия начиналась только спустя месяц после РЙТ, поэтому связь между гипотиреозом и аменореей авторами была исключена. При поиске предикторов изменения функции яичников не было выявлено корреляции между приемом комбинированных оральных контрацептивов или аутоиммунными заболеваниями ЩЖ. При сравнении женщин с нарушением МЦ и без него значимых различий между введенной активностью (10,3±2,0 против 9,8±2,3 ГБк), поглощенной дозой или дозой облучения яичников (1,76±0,49 против 1,58±0,43 Гр) установлено не было. Возраст стал единственным предиктором развития аменореи — чем старше была женщина, тем выше риск нарушения МЦ. Средний возраст пациенток с аменореей составил 38,9±7,1 года в сравнении с 32,2±6,1 года у женщин без нарушений МЦ (P<0,001). Только в группе женщин 45–49 лет у 9 из 26 наступила менопауза [28]. По нашему мнению, кроме возможной индивидуальной чувствительности яичников к облучению, эти наблюдения можно объяснить возрастными изменениями в яичниках, а именно атрезией фолликулов и снижением овариального резерва, которые в условиях проведения РЙТ и дисрегуляции гипоталамо-гипофизарно-яичниковой оси могут усугубляться. Позднее Izembart и коллеги рассчитали дозу ¹³¹I, получаемую яичником, с целью создания дозиметрической модели, однако, как отмечают сами авторы, модель была неточна. Было выдвинуто предположение, что помимо дозы морфологические и кинетические параметры, а также индивидуальные характеристики, такие как возраст, могут иметь значение [29].

Основываясь на этих еще малочисленных и малоинформативных исследованиях, в 1995 г. в «Журнале ядерной медицины» (Journal of Nuclear Medicine) вышла редакционная статья, декларирующая отсутствие какого-либо значимого влияния РЙТ на репродуктивное здоровье женщин с ДРЩЖ [30].

Одними из первых работ, оценивающих влияние РЙТ на фертильность пациентов с ДРЩЖ, стали исследования Sarkar и соавт. и Edmonds и соавт., проведенные в 1976 г. и 1986 г. соответственно. В первое исследование было включено 33 пациентки с ДРЩЖ, получивших РЙТ в детском и подростковом возрасте. Оказалось, что даже при высокой кумулятивной дозе частота таких осложнений, как бесплодие, выкидыш, недоношенность и врожденные пороки развития плода, существенно не отличалась от таковой в общей популяции и составила 12, 1,4, 8 и 1,4% соответственно [31]. В этой же работе рассмотрена связь изучаемого репродуктивного анамнеза с возрастом, когда была начата терапия, кумулятивной дозой и периодом наблюдения. Значимых различий между пациентками с осложнениями и без обнаружено не было: средний возраст 9 пациенток с осложнениями составил 16,3±3,7 года, у 24 пациенток без — 14,0±4,1 года (Р>0,10), средняя кумулятивная активность у пациентов с осложнениями составила 224±184 мКи по сравнению с 186±112 мКи у пациентов без осложнений; (Р>0,40), средняя продолжительность наблюдения в группах с осложнениями и без них составила 19,6±4,2 года и 18,4±3,1 года соответственно. В исследовании Edmonds по развитию долгосрочных осложнений РЙТ изучалась рождаемость у пациентов репродуктивного возраста с ДРЩЖ, которые состояли в браке и планировали беременность. Только 4 (13%) из 31 пациентов не смогли реализовать свои репродуктивные планы после РЙТ. Не было установлено корреляции между дозой ¹³¹I и снижением фертильности, однако отмечено, что может существовать индивидуальная предрасположенность к развитию данного осложнения [32].

Smith и соавт. также оценивали долгосрочное влияние ¹³¹I на фертильность пациенток, прошедших РЙТ в детском и подростковом возрасте. Средний период наблюдения составил 16,8 года, средняя введенная активность — 148 мКи. Полученные результаты соотносились с исследованием Sarkar: риск бесплодия у женщин с ДРЩЖ, прошедших РЙТ, не отличался от такового в общей популяции, также как и риск врожденных пороков развития у потомства в случае, если беременность наступала не ранее чем через год после РЙТ [33].

Еще одно ретроспективное исследование, проведенное Dottorini и соавт. в 1995 г., включало 627 женщин с ДРЩЖ, прошедших РЙТ и получавших супрессивную терапию (1 группа), и 187 женщин с ДРЩЖ, прошедших только ТЭ и получавших заместительную терапию (2 группа). Средний период наблюдения составил 4,5 года в первой группе и 6,4 года во второй, средняя активность составила 5,4 ГБк в обеих группах. Фертильность оценивалась путем регистрации количества родов, дополнительно изучались исходы беременности. По итогу значимых различий в рождаемости, а также различий в массе тела новорожденных и частоте недоношенности между двумя группами не было (P=0,39) [34].

Безусловно, эти ранние работы в силу отсутствия точных методов оценки функции яичников и фертильности не смогли прояснить, как РЙТ влияет на репродуктивный потенциал, однако они стали первыми шагами в этом направлении и показали, что рождаемость в группе пациентов, получавших РЙТ по поводу ДРЩЖ, а также частота осложнений беременности значимо не отличается от общей популяции при условии соблюдения рекомендаций по планированию беременности после РЙТ.

## СОВРЕМЕННЫЙ ВЗГЛЯД НА ВОПРОС О ВЛИЯНИИ РЙТ НА РЕПРОДУКТИВНУЮ ФУНКЦИЮ ЖЕНЩИН С ДРЩЖ

Яичники не накапливают ¹³¹I , однако могут получать дозу облучения из крови и в меньшей степени — от близлежащих органов, участвующих в выведении ¹³¹I, а именно мочевого пузыря и толстого кишечника [6]. Предполагается, что воздействие ¹³¹I на яичники, в особенности на фолликулярный аппарат, может приводить к развитию ДЯ, изменению качества и количества фолликулов и сокращать репродуктивный период.

В ретроспективном исследовании Ceccarelli и соавт. впервые было показало, что РЙТ может привести к более раннему наступлению менопаузы — 49,5 года после РЙТ по сравнению с 51 у пациенток, не получавших РЙТ (P<0,001). Стоит отметить, что в данном исследовании не проводился гормональный анализ, авторы оценивали наличие/отсутствие клинических проявлений менопаузы (в то время она определялась как отсутствие менструаций в течение не менее 6 месяцев подряд). Была выдвинута гипотеза, что более раннее наступление менопаузы также может быть следствием стресса, возникающего на фоне онкологического заболевания, или аутоиммунных заболеваний ЩЖ, а не только из-за непосредственного воздействия ¹³¹I [35]. В 2006 г. опубликовано исследование с похожими результатами: Souza Rosario и соавт. наблюдали достоверно более раннее наступление менопаузы у женщин, получивших РЙТ — 46 лет после РЙТ по сравнению с 50,4 года у пациенток, не получавших РЙТ (P<0,001). Все пациентки после первоначального лечения получали супрессивную терапию, и уровень супрессии ТТГ не отличался между двумя группами. В отличие от предыдущих исследований, было отмечено влияние дозы ¹³¹I — возраст менопаузы у 36 женщин, получавших активность 100 мКи, составил 48 лет по сравнению с 45 годами у женщин, получивших ¹³¹I активностью >100 мКи (P<0,003). У пациенток, получавших активность 100 мКи, менопауза наступала достоверно раньше, чем у тех, кто не проходил РЙТ (P<0,03). У 33% пациенток контрольной группы в возрасте 51 года сохранялся МЦ, тогда как в основной только в 16% [36]. В ретроспективном исследовании Manuel García-Quirós Muñoz также анализировалось влияние РЙТ на функцию яичников у 202 женщин с ДРЩЖ. У пациенток, получивших более 200 мКи, менопауза наступила раньше, чем у пациенток, получивших менее 200 мКи (р<0,01), однако при сравнении возраста менопаузы у пациенток после РЙТ с таковым у их родственников первой степени родства, матерей и сестер, статистически значимой разницы не обнаружено [37].

Транзиторные нарушения МЦ по типу олигоменореи/аменореи могут наблюдаться в 8–31% случаев в течение первых 10–12 месяцев после РЙТ, при этом, кроме самой РЙТ, подавление уровня ТТГ после лечения может быть одной из причин развития данного осложнения (таблица 1) [38–43].

**Table:** Таблица 1. Функция яичников у женщин с ДРЩЖ, прошедших РЙТ

Источник, год	Выборка	Доза ¹³¹I	Эффект РЙТ (результат исследования)	Вывод
Ceccarelli и соавт. 2001 г. [35]	Основная группа — 130 Контрольная группа — 127	1,11–40,7 ГБк (кумулятивная активность)	Средний возраст менопаузы в основной группе — 49,5 года по сравнению с 51 годом в контрольной (P<0,001)	РЙТ может приводить к более раннему наступлению менопаузы
Esfahani и соавт. 2004 г. [41]	159	≥3,7 ГБк	Отсутствие нарушений МЦ и значимых изменений в уровнях ЛГ, ФСГ, эстрогена и прогестерона после РЙТ (Р — NS)	Не выявлено влияния на функцию яичников
Souza Rosario и соавт. 2006 г. [36]	Основная группа — 74 Контрольная группа (пациенты с ДРЩЖ, не получающие РЙТ) — 40	3,7–7,4 ГБк	Средний возраст менопаузы в основной группе — 46 лет по сравнению с 50,4 года в контрольной (P<0,001)	РЙТ может приводить к более раннему наступлению менопаузы
Vini и соавт. 2002 г. [38]	496	3–59 ГБк	Аменорея в 8% случаев, другое нарушение МЦ — в 12%	РЙТ может приводить к транзиторному нарушению МЦ
Souza Rosario и соавт. 2005 г. [39]	50	3,7–5,5 ГБк	У 20% пациентов — аменорея. Уровень ФСГ повысился спустя 6 мес после РЙТ (P<0,00001). У 28% пациентов — повышение уровня ФСГ более 15 МЕ/л	РЙТ может приводить к транзиторному нарушению МЦ и повышению уровня ФСГ
Sioka и соавт. 2006 г. [40]	Основная группа — 45 Контрольная группа (женщины без патологии ЩЖ) — 83	3,7 ГБк	В основной группе нарушения МЦ после РЙТ наблюдались в 31,1% случаев по сравнению с 14,5% в контрольной (P<0,02)	РЙТ может приводить к нарушению МЦ
Manuel García-Quirós Muñoz и соавт. 2010 г. [37]	Основная группа — 34 Контрольная группа — матери и сестры пациенток	≥3,7 ГБк	При активности менее 200 мКи средний возраст составил 50,42±3,62, при активности более 200 мКи — 47,66±2,31 (P<0,01). Средний возраст менопаузы составил 49,94±3,45 года, что соотносится с возрастом наступления менопаузы у их родственников первой линии родства, однако менопауза наступала раньше, чем в общей популяции (51,7 года)	РЙТ не сокращает репродуктивный период при сопоставлении среднего возраста менопаузы у пациенток с ДРЩЖ с таковым у их родственников первой линии родства
Acıbucu и соавт. 2016 г. [42]	Основная группа — 45 Контрольная группа (здоровые женщины) — 40	3,7–5,55 ГБк	Олигоменорея в течение 3–5 мес в 15,6% после РЙТ	РЙТ может приводить к транзиторному нарушению МЦ
Adamska и соавт. 2021 г. [43]	25	3,7 ГБк	Нарушение менструального цикла в 16% в течение первого года после РЙТ	РЙТ может приводить к транзиторному нарушению МЦ

Сейчас существуют различные диагностические маркеры, позволяющие более точно оценить репродуктивную функцию женщины на фоне терапии ДРЩЖ. В 1971 г. Josso, французский детский эндокринолог, впервые выделила антимюллеров гормон (АМГ), гликопротеин суперсемейства трансформирующих факторов роста P (TGF-P), играющий важную роль в формировании мужского пола у плода. Позднее Josso совместно с Vigier установила, что АМГ синтезируется гранулезными клетками преантральных и малых антральных фолликулов у женщин репродуктивного возраста [44]. В настоящее время АМГ — один из чувствительных маркеров овариального резерва (ОР) и ранний маркер его возрастного снижения, позволяющий судить о репродуктивном периоде и времени наступления менопаузы [45]. Он используется в программах экстракорпорального оплодотворения для прогнозирования ответа яичников, при диагностике ПНЯ и патологических состояний — синдроме поликистозных яичников, опухолей гранулезных клеток. АМГ также применяют для оценки гонадотоксичности различных методов лечения, особенно при онкологических заболеваниях [46]. У девочек при рождении АМГ практически не определяется, он начинает расти к пубертату и сохраняется на стабильном уровне в раннем репродуктивном периоде, после 30 лет АМГ постепенно снижается до необнаруживаемых значений к менопаузе [47].

В 2016 г. Acibucu и соавт. впервые использовали АМГ для оценки влияния РЙТ на ОР пациенток с ДРЩЖ. Были установлены более низкие средние значения АМГ у женщин, получивших РЙТ, по сравнению с контрольной группой (Р<0,038) [42]. В 2018 г. в Израиле было проведено проспективное исследование, оценивающее АМГ у женщин с ДРЩЖ в динамике — до РЙТ, каждые 3 месяца в течение первого года после лечения. Оно включало 24 женщины репродуктивного возраста с ДРЩЖ (средний возраст — 34 года; диапазон — 20–45 лет), которым планировалось проведение первого курса РЙТ. По результатам, произошло значительное снижение концентрации АМГ через 3 месяца после РЙТ (P<0,0001) с лишь частичным восстановлением. Предикторами снижения АМГ через 3 месяца были возраст — у женщин старше 35 лет достоверно чаще наблюдалось выраженное снижение АМГ через 3 месяца (63,7±18,5% против 33,1±29,2%; P=0,01) и более раннее наступление менархе (P=0,03). Тиреоидит Хашимото (ТХ) также имел связь со степенью снижения уровня АМГ через 3 месяца — у 5 пациенток с ТХ снижение составило 72% по сравнению с 42% при отсутствии данного диагноза (P=0,015). При этом исходный уровень АМГ не отличался у пациенток с ДРЩЖ и ТХ и у пациенток с ДРЩЖ без ТХ — 3,36±1,7 нг/мл и 3,22±0,59 нг/мл соответственно. Контрольной группой в данном исследовании были пациентки с БГ, чей уровень АМГ значимо не изменялся и составил 2,6±2,0 нг/мл исходно и 3,3±2,35 нг/мл, 3,6±2,14 нг/мл, 3,3±2,52 нг/мл и 3,4±1,83 нг/мл через 3, 6, 9 и 12 мес соответственно [48].

Влияние РЙТ на ОР изучалось Evranos и коллегами в 2018 г. По результатам исследования, у женщин с ДРЩЖ уровень АМГ был выше до РЙТ (P=0,001), а уровни ФСГ, ЛГ и эстрадиола значимо не менялись (P>0,05) [49]. Уровень АМГ в течение длительного периода времени (в среднем 34 месяца) исследовался в работе van Velsen и соавт. У пациенток, получивших один курс РЙТ, уровень АМГ снижался нелинейно в среднем на 55% в течение первых 12 месяцев и стабилизировался после этого. У женщин, прошедших более одного курса РЙТ, наблюдалось дальнейшее снижение АМГ (на 85% через 48 месяцев). Возраст коррелировал со снижением АМГ, при этом у более молодых пациенток (<35 лет) наблюдалось менее резкое снижение [50].

В исследовании Navarro и соавт. было установлено, что РЙТ может повлиять на репродуктивную функцию: 17,9% женщин столкнулись с трудностями при зачатии ребенка, а у 40% женщин (4 из 10), прошедших РЙТ, наступила ранняя менопауза, однако значимых изменений в уровне АМГ зафиксировано не было [51]. В проспективном исследовании Hosseini, опубликованном в 2023 г., средний уровень АМГ через 3, 6 и 12 месяцев после РЙТ достоверно снизился по сравнению с показателями до (Р<0,001) [52].

Кроме АМГ для оценки ОР могут использовать ингибин В, подсчет количества антральных фолликулов (КАФ), а также ФСГ. Все эти параметры были изучены Adamska и соавт. — у пациенток с ДРЩЖ обнаружено снижение КАФ (P=0,03), уровня АМГ в сыворотке крови (P<0,01), ингибина B (P=0,03) через 1 год после РЙТ по сравнению с исходными значениями, при этом уровень ФСГ значимо не отличался [43].

Таким образом, в большинстве проведенных за последние годы исследований отмечено значимое влияние РЙТ на ОР, определяемое прежде всего путем оценки уровня АМГ до и после терапии (таблица 2).

**Table:** Таблица 2. Овариальный резерв у женщин с ДРЩЖ, прошедших РЙТ

Источник, год	Выборка	Доза ¹³¹I	Эффект РЙТ	Вывод
Acıbucu и соавт. 2016 г. [42]	Основная группа — 45 Контрольная группа (здоровые женщины) — 40	3,7–5,55 ГБк	Средний уровень АМГ после РЙТ составил 2,50 по сравнению с 2,92 в контрольной группе (Р<0,038)	РЙТ влияет на ОР
Yaish и соавт. 2018 г. [48]	Основная группа — 24 Контрольная группа (пациентки с БГ) — 5	1,11–5,55 ГБк (средняя — 3,8) 0,48 ГБк	В группе с ДРЩЖ уровень АМГ до РЙТ составил 3,25±0,56 нг/мл, а после РЙТ значимо снизился: через 3 мес — 1,9±0,38 нг/мл (Р<0,001), через 6 мес — 2,23±0,43 нг/мл (Р<0,01), через 9 мес — 2,47±0,47 нг/мл (Р<0,05), через 12 мес — 2,36±0,47 нг/мл (Р<0,05) В контрольной группе значимой разницы в уровне АМГ до и после РЙТ не обнаружено (Р — NS)	РЙТ влияет на ОР
Evranos и соавт. 2018 г. [49]	33	2,8–5,55 ГБк	Уровень АМГ снизился с 3,25 нг/мл до 1 нг/мл, 1,13 нг/мл и 1,37 нг/мл через 3, 6, 12 мес соответственно и значимо не менялся в течение первого года после РЙТ	РЙТ влияет на ОР
van Velsen и соавт. 2020 г. [50]	65	47 пациенток прошли 1 курс РЙТ активностью 1,11–5,4 ГБк 18 пациенток — более 1 курса, суммарная активность — 10,5–10,8 ГБк	У пациенток, получивших 1 курс РЙТ, уровень АМГ до лечения составил 3,05±1,21 нг/мл, через год — 1,38±1,2 нг/мл (снижение на 55%, P<0,0001). У пациенток, получивших более 1 курса РЙТ, АМГ до лечения составил 3,37±1,54 нг/мл, через год — 0,86±1,48 нг/мл (снижение на 74%, P<0,001). Через 2 года после лечения уровень АМГ был ниже во второй группе (P=0,005)	РЙТ влияет на ОР, особенно у пациенток, прошедших несколько курсов РЙТ
Adamska и соавт. 2021 г. [43]	25 пациенток, из них 11 пациенток старше 35 лет, 14 пациенток — младше	3,7 ГБк	Средний уровень АМГ до РЙТ составил 2,7 нг/мл и 1,0 нг/мл (P=0,01) у женщин младше и старше 35 лет соответственно, через год после РЙТ — 2,4 нг/мл и 0,5 нг/мл (P=0,01). При сравнении женщин младше 35 лет и старше выявлено значимое снижение уровня АМГ в обеих группах (P=0,04 и P<0,01, соответственно)	РЙТ влияет на ОР
Navarro и соавт. 2022 г. [51]	Основная группа — 40. Контрольная группа (пациентки с ДРЩЖ, прошедшие только ТЭ) — 11	1–13,4 ГБк	После РЙТ средний уровень АМГ составил 1,18±1,2 нг/мл, при этом в контрольной группе — 1,24±1,0 (P=0,692). В основной группе АМГ был у 29,2% ниже нормальных значений, а в контрольной — у 11% (Р — NS)	Нет значимых различий в уровне АМГ между пациентками с ДРЩЖ, получавшими и не получавшими РЙТ, однако 17,5% женщин, прошедших РЙТ, столкнулись с трудностями при зачатии
Hosseini и соавт. 2023 г. [52]	60	3,7 ГБк	Уровень АМГ до РЙТ — 2,25±0,55 нг/мл, после — 1,15±0,35 нг/мл (на 49,05% ниже исходного), 1,58±0,47 нг/мл (на 29,55% ниже исходного) и 1,94±0,58 нг/мл (на 13,58% ниже исходного) через 3, 6 и 12 мес после РЙТ соответственно (P<0,001)	РЙТ влияет на ОР

## ОБСУЖДЕНИЕ

Несмотря на благоприятный прогноз, ДРЩЖ сопряжен со снижением качества жизни, в особенности у пациентов, получающих комбинированное лечение. Это может влиять на репродуктивные планы пациентов и приводить к более позднему наступлению беременности и снижению рождаемости [5][53][54]. Основные причины: рекомендации врачей отсрочить беременность (как, например, в группе пациентов, получающих РЙТ), страх перед зачатием на фоне онкологического заболевания, а также непосредственное влияние проводимого лечения на репродуктивную функцию.

Опытным путем доказано, что яичники не накапливают ¹³¹I, но могут получать дозу облучения из крови и близлежащих органов, а также метастатических очагов, захватывающих ¹³¹I и располагающихся в малом тазу. При этом клетки, которые активно делятся, более подвержены действию радиации, а значит, растущие фолликулы уязвимы к действию ¹³¹I. Если способом подготовки к РЙТ становится отмена тиреоидных гормонов, то на момент терапии пациенты находятся в состоянии гипотиреоза, который в свою очередь способствует снижению почечного клиренса ¹³¹I и его задержке в организме, повышая риск возникновения вторичных осложнений, в том числе со стороны репродуктивной системы. На основании проведенных исследований в этой области оказалось, что вероятность нарушения МЦ повышается с возрастом и, как в случае других вторичных осложнений, с дозой ¹³¹I (от 3,7 ГБк), хотя ДЯ развивается и у молодых пациенток, получающих более низкие активности (до 3,7 ГБк), и может быть связана с индивидуальными особенностями и/или наличием дополнительных факторов риска [28][38–40][42][43][48][55]. Cмена тиреоидного статуса на различных этапах лечения с чередованием эутиреоза, гипотиреоза и субклинического тиреотоксикоза может влиять на функциональное состояние гипоталамо-гипофизарно-яичниковой оси, а супрессивная терапия после РЙТ — одна из возможных причин ДЯ [42][56]. Однако нарушения МЦ могут наблюдаться и в случаях, когда пациентки получают заместительную терапию. Олигоменорея/аменорея, как правило, носит транзиторный характер и наблюдается до года после РЙТ и менее чем в половине случаев [40][42][48]. Кроме того, РЙТ может сокращать репродуктивный период — у женщин, получивших РЙТ по поводу ДРЩЖ, менопауза наступала раньше, чем у тех, кто не проходил РЙТ (контрольная группа), и/или раньше, чем в общей популяции, при этом в исследованиях Souza Rosario и Manuel García-Quirós Muñoz этот эффект был дозозависим, а в исследовании Navarro и соавт. в 40% случаев развивалась ранняя менопауза [35–37][51][55].

Значимое изменение уровня АМГ (в ряде случаев — на 55% от исходного уровня, а также опережающее возрастное снижение) после РЙТ отмечалось в большинстве работ, что позволяет рекомендовать женщинам репродуктивного возраста оценивать ОР перед лечением для решения вопроса о необходимости применения мер по сохранению фертильности, для этого может использоваться или определение АМГ, или комбинация методов, предпочтительно определение АМГ и КАФ [42][43][48–52][57][58]. Это особенно важно, если диагноз «ДРЩЖ» установлен до наступления первой беременности, при наличии гинекологических заболеваний, влияющих на фертильность, а также при изначально или ожидаемо сниженном уровне АМГ, как в случае возрастных пациенток или пациенток с бесплодием, планирующих беременность. Что касается предикторов снижения ОР после РЙТ, включая и вероятность снижения АМГ как основного маркера ОР, и его степень, то их еще предстоит определить. В настоящее время показано, что снижение уровня АМГ после РЙТ отмечается независимо от их возраста, однако у пациенток старше 35 лет оно более существенно [42][48–50]. Следующим фактором риска потенциально может стать получаемая активность и количество курсов РЙТ, как и в случае других вторичных осложнений, однако в большинстве проведенных исследований корреляции установлено не было [48–50][59]. В исследовании Yaish и соавт. снижение АМГ у пациенток с ДРЩЖ отмечено и при активности в 1,11 ГБк, в сравнении с этим у пациенток с тиреотоксикозом, получающих как правило значимо более низкие активности (менее 1,11 ГБк), отсутствовало влияние РЙТ на репродуктивную функцию [48]. В проспективном исследовании van Velsen и соавт. пациентки, прошедшие РЙТ более 1 раза, имели более значимое снижение уровня АМГ (на 74% от исходных значений), что может быть связано как с большей кумулятивной активностью, так и с многократным изменением тиреоидного статуса [50].

В начале 2000-х, с одобрением рчТТГ в качестве метода подготовки к радионуклидной диагностике и РЙТ, появилась возможность снизить лучевую нагрузку на организм пациента, что в свою очередь способствует снижению риска ряда вторичных осложнений РЙТ [10][60]. Действительно, использование рчТТГ ассоциируется с меньшей частотой поражения слюнных и слезных желез, однако с большим процентом облитерации слезоотводящих путей [61–64]. Вопрос о влиянии метода подготовки к послеоперационной РЙТ на репродуктивную систему женщины с ДРЩЖ остается открытым — нами не было найдено работ, анализирующих этот аспект. Так, в проспективном исследовании Adamska и соавт., отмечалось нарушение МЦ в 15,6% случаев и снижение маркеров ОР, в особенности АМГ, при введении рчТТГ в качестве подготовки к РЙТ, однако сравнительная группа на отмене тиреоидных гормонов отсутствовала [43]. Похожие наблюдения относительно снижения уровня АМГ были сделаны в работе Yaish и соавт. [48]. Наблюдаемые изменения ОР соотносятся с результатами работ, в которых в качестве метода повышения ТТГ перед РЙТ использована отмена тиреоидных гормонов [49][52].

Опираясь на результаты приведенных исследований, мы предполагаем, что потенциальными факторами риска изменения репродуктивной функции у пациенток, проходящих комбинированное лечение по поводу ДРЩЖ, могут стать возраст, кумулятивная активность, неоднократная смена тиреоидного статуса, степень подавления ТТГ, метод подготовки к РЙТ, наличие сопутствующего аутоиммунного заболевания ЩЖ, наличие вредных привычек и индивидуальные особенности акушерско-гинекологического анамнеза.

Стоит отметить, что изменение функции яичников, характеризующееся транзиторными нарушениями МЦ, а также снижение ОР после РЙТ, определяемое по уровню АМГ и другим биохимическим и инструментальным маркерам, не отражает качество ооцитов и не равно снижению фертильности. В последние годы было проведено много исследований, оценивающих репродуктивную функцию женщин с ДРЩЖ, получающих РЙТ, однако часть из них носят ретроспективный характер, а часть имеет небольшие выборки, непродолжительный период наблюдения и исследует ограниченные параметры, что затрудняет адекватный анализ изменений, происходящих в женской репродуктивной системе. С целью установки истинного и долгосрочного влияния РЙТ на репродуктивную функцию требуются более крупные контролируемые проспективные исследования с периодом наблюдения от нескольких лет, включающие пациенток как с однократной, так и с многократной РЙТ и получающих различную подготовку к РЙТ, что позволит определить предикторы ДЯ репродуктивного возраста, более раннего наступления менопаузы и снижения ОР, а также установить группы риска по развитию данных осложнений.

Безусловно, направление пациентов на РЙТ должно реализовываться в соответствии с настоящими рекомендациями, избегая случаев назначения такого лечения, когда оно не имеет клинического значения, например, при низком риске рецидива, а в случае комбинированного лечения ДРЩЖ пациенток репродуктивного возраста необходимо информировать о возможных осложнениях РЙТ и рекомендовать оценивать ОР и своевременно направлять на консультацию акушера-гинеколога и/или репродуктолога.

## ЗАКЛЮЧЕНИЕ

В настоящее время до 30% пациенток сталкиваются с транзиторным нарушением МЦ после РЙТ, более чем в половине случаев наблюдается снижение ОР. Однако какова клиническая значимость данных изменений и насколько это отражается на репродуктивном здоровье в долгосрочной перспективе, до конца неясно. Кроме того, РЙТ ассоциирована с более ранним наступлением менопаузы — в среднем на 1,5–5 лет раньше, чем у женщин, не получавших такое лечение.

В настоящее время, учитывая важность персонализированного подхода к лечению, прогнозирование возможных рисков вторичных осложнений, в том числе репродуктивных нарушений на фоне терапии ДРЩЖ, крайне актуально, особенно учитывая снижение рождаемости и увеличение числа бесплодных пар в общей популяции. Знания о рисках, ассоциированных с РЙТ, позволят клиницистам своевременно принимать решение о тактике ведения пациентов с ДРЩЖ, необходимости применения мер по профилактике осложнений и проведении мероприятий, направленных на сохранение фертильности.

## ДОПОЛНИТЕЛЬНАЯ ИНФОРМАЦИЯ

Источники финансирования. Исследование выполнено в рамках государственного задания № 123021000041-6.

Конфликт интересов. Авторы декларируют отсутствие явных и потенциальных конфликтов интересов, связанных с содержанием настоящей статьи.

Участие авторов. Авторы декларируют соответствие своего авторства международным критериям ICMJE. Все авторы внесли равный вклад в подготовку статьи и одобрили ее окончательный вариант.
